# Hereditary Spherocytosis

**DOI:** 10.3329/jhpn.v28i1.4529

**Published:** 2010-02

**Authors:** Sayeeda Huq, Mark A.C. Pietroni, Hafizur Rahman, Mohammad Tariqul Alam

**Affiliations:** ^1^ Clinical Sciences Division; ^2^ Dhaka Hospital; ^3^ Laboratory Sciences Division, ICDDR,B, GPO Box 128, Dhaka 1000, Bangladesh

**Keywords:** Anaemia, Anaemia, Haemolytic, Spherocytosis, Hereditary, Bangladesh

## Abstract

A 12-year old girl was brought to the Dhaka Hospital of ICDDR,B with diarrhoea. Incidentally, the parents provided a history of repeated episodes of pallor and jaundice since she was two and half years old. Three of her family members had similar problems. History, clinical examination, and laboratory findings of the girl and her family members suggested a case of hereditary spherocytosis. To our knowledge, this is the first report of such a case in Bangladesh.

## INTRODUCTION

Hereditary spherocytosis (HS) is a familial haemolytic disorder with marked heterogeneity. Clinical features range from asymptomatic to fulminant haemolytic anaemia. The condition was first described in 1871 (1). HS is the commonest cause of inherited chronic haemolysis in Northern Europe and North America, with a reported incidence of 1 in 5,000 births (2). In 80% of the instances, the inheritance of HS is autosomal dominant and in others autosomal recessive (3). In North India, both autosomal dominant and recessive patterns have been reported with presentation similar to that in other populations but the underlying protein defect has not been characterized (4). To our knowledge, no case has been reported from Bangladesh.

A family history and typical clinical and laboratory findings make the diagnosis possible without much difficulty, and additional investigations are not required. Atypical cases may require measurement of erythrocyte membrane proteins to clarify the nature of the membrane disorder, and occasionally, molecular genetic tests are required to determine the mode of inheritance. In autosomal dominant form, the deficiency is mild, and hence, the anaemia is mild while in the recessive form, the deficiency is greater, and the anaemia is profound. (2)

### Case summary

In May 2009, a 12-year old girl was brought to the Dhaka Hospital of ICDDR,B and was admitted with a three-day history of diarrhoea, high-grade fever, abdominal pain, and weakness. The girl's family lives in Dhaka city, and her parents are non-consanguineous. Before attending the hospital, the child was treated with some unspecified medicines. Systemic enquiry revealed that she had been suffering from repeated attacks of jaundice and pallor since she was 2.5 years old. This long-standing illness was usually managed by homeopathic medicines and those prescribed by ‘quacks’ but she was occasionally given folic acid. Her birth history was uneventful, and she did not have neonatal jaundice. The girl was immunized as per the Expanded Programme on Immunization schedule.

Among family members, her father, one brother, and one sister have similar problems, and they were asked to attend the hospital. They all provided a history of breathlessness on exertion, puffiness of face, abdominal pain, yellow colouration of sclera, and pallor.

At admission, the girl looked sick, was pale, and had icteric sclerae. She was afebrile. Her pulse rate was 110 per minute, blood pressure was 100/70 mm Hg, and she had a systolic flow murmur on auscultation. Her respiration rate was 22 per minute. On abdominal examination, her liver was palpable 2 cm below the right costal margin in the mid-clavicular line and was soft and non-tender. Her spleen was palpable 4 cm below left costal margin along the long axis on deep inspiration and was soft in consistency. Other systemic examinations did not reveal any abnormality. A provisional diagnosis of acute watery diarrhoea, congenital haemolytic anaemia, and flow murmur due to long-standing anaemia was made.

**Fig. 1.**
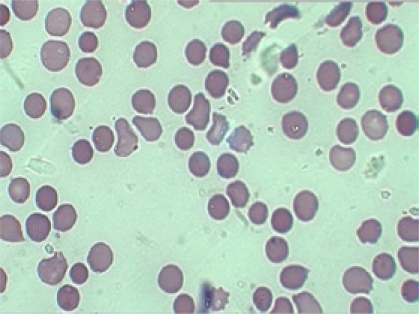
Spherocytes

Her haemoglobin and haematocrit on admission were 11.3 g/L and 31.6% respectively, with a peripheral total white blood cell count of 12,900/mm^3^ with unremarkable differential counts. The mean corpuscular volume (MCV) was 82.8 fL, the mean corpuscular haemoglobin (MCH) was 30.7 pg, the mean corpuscular haemoglobin concentration (MCHC) was 37.1 g/dL, and the reticulocyte count was 11.0%. Serum electrolytes were within normal limits. Liver function tests were abnormal with a raised lactate dehydrogenase (LDH) at 302.4 U/L, and a raised total bilirubin was 127 μmol/L. Further investigations showed a normocytic, normochromic peripheral blood film with significant spherocytes (Fig. 1). Haemoglobin electrophoresis was normal. Coombs test—both direct and indirect—was negative. In an osmotic fragility test of red blood cell (RBC), lysis started at 0.7% saline and was completed at 0.3% saline; 50% mean corpuscular fragility was obtained at 0.5% saline. Blood and rectal swab cultures did not grow any pathogen. Ultrasonographic examination of her abdomen revealed hepatosplenomegaly, and gallstones were not found.

During her hospital stay, the patient received oral rice-water saline for dehydration from diarrhoea, folic acid, and multivitamins.

### Differential diagnosis

**Fig. 2.**
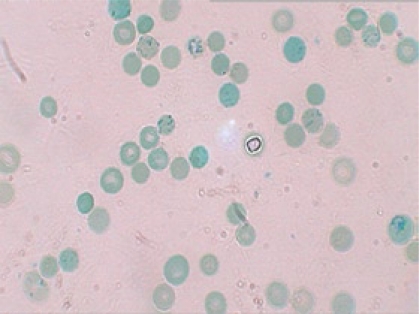
Reticulocytes

In the presence of a large number of spherocytes in the peripheral blood film, a major alternative differential diagnosis was autoimmune haemolysis (which can mimic HS). A positive direct Coomb's test (detection of antibody on RBCs using direct antiglobulin) indicates autoimmune haemolytic anaemia. Other rare causes of spherocytosis include thermal injury, clostridial septicaemia with exotoxaemia, and Wilson's disease, which may present with transient haemolytic anaemia (2). Haemolytic anaemia is also a feature of haemoglobinopathies, which are diagnosed by haemoglobin electrophoresis. In HS, the electrophoresis is normal, and the presence of microspherocytes on the blood smear confirms the diagnosis. The differentials focus on the causes of a fall in the RBC surface area/volume that occur in autoimmune haemolytic anaemia, HS, and microangiopathic anaemia. However, in our presented case, there were no suggestive features, such as fragmented RBC, in favour of microangiopathy. Therefore, a negative direct Combs test, positive osmotic fragility test, a blood smear showing spherocytes, and a raised reticulocyte count (Fig. 2) all suggest that hereditary spherocytosis was the likely diagnosis.

## DISCUSSION

Microspherocytosis is the morphological hallmark of HS, which is caused by loss of membrane surface area, with abnormal osmotic fragility *in vitro*. An intrinsic genetic defect causes defects in spectrin, an RBC membrane protein, which are the major components of the cytoskeleton responsible for maintaining the shape of the RBCs. Haemolysis in HS results from the relationship of an intact spleen and an intrinsic membrane protein defect that leads to abnormal RBC morphology. In general, HS caused by α-spectrin and β-spectrin mutations represent recessive and dominant inheritance respectively. Patients with β-spectrin deficiency typically have mild to moderately-severe disease and do not need transfusion (2).

HS may manifest as haemolytic disease in the newborns and may present with anaemia and hyperbilirubinaemia. Although some children are asymptomatic in childhood, others may have pallor (anaemia), fatigue, and exercise intolerance. In severe cases, marked expansion of the diploe skull-bones and medullary region of other bones is observed but to a much lesser extent than observed in patients with thalassaemia. The spleen is enlarged after infancy, and pigmented gallstones may develop as early as 4–5 years.

HS is usually diagnosed clinically from a typical family history and splenomegaly and confirmed by the presence of many spherocytes and reticulocytes in the peripheral blood film. The presence of spherocytes is confirmed by an osmotic fragility test, the sensitivity of which varies widely from 48% to 95%, although this may be increased to 99% by the glycerol lysis test and the NaCl test on incubated blood (5).

Haemolytic crisis and megaloblastic crisis are the most common complications of HS. High RBC turnover and heightened erythroid marrow activity in HS make children vulnerable to develop aplastic crisis due to parvovirus and various other infections. Erythroid marrow failure may result in profound anaemia, heart failure, hypoxia, cardiovascular collapse, and death.

Spherocytes in HS are destroyed solely in the spleen, and, therefore, haemolysis may be prevented by splenectomy. Splenectomy often improves osmotic fragility due to diminished splenic conditioning and reduced loss of the RBC membrane. However, splenectomy carries significant risks and, for HS, is not recommended in patients with haemoglobin exceeding 10 g/dL and a reticulocyte count of less than 10%. When splenectomy is indicated, which is usually done in patients aged at least 5–6 years, administration of pneumococcal, *Haemophilus influenzae* type b, meningococcal group C, and influenza vaccines 2–3 weeks before the surgery is recommended. Patients should be given 1 mg of folic acid daily for preventing secondary folic acid deficiency, and oral penicillin (penicillin V) for preventing secondary infection until reaching adulthood. Since patients are more prone to haemolysis, a bracelet or card indicating the diagnosis should be worn to alert health professionals.

HS, as the name suggests, is inherited and can pass down from parents to children. Families with an affected child should be counselled about up to 50% probability of each subsequent child having HS. Although genetic counselling is difficult to do in most developing countries due to the non-availability of genetic testing, HS is a relatively-straightforward clinical diagnosis of a genetic condition; so, parents have the opportunity to receive counselling about the consequences of the diagnosis, the prognosis, and the risk of another child being affected.
